# Retrospective Italian Registry on DSM-TACE: Experience Beyond Current Recommendations

**DOI:** 10.3390/cancers18050736

**Published:** 2026-02-25

**Authors:** Pierleone Lucatelli, Maria Giulia Travaglini, Elio Damato, Francesco Giurazza, Anna Maria Ierardi, Giacomo Luppi, Michele Citone, Roberto Cianni, Gianluca De Rubeis, Pierpaolo Biondetti, Fabio Corvino, Claudio Carrubba, Giulio Vallati, Federico Cappelli, Alessandro Posa, Marcello Lippi, Mario Corona, Valeria Panebianco, Carlo Catalano, Roberto Iezzi

**Affiliations:** 1 Vascular and Interventional Radiology Unit, Department of Radiological, Oncological and Pathological Sciences, Sapienza University of Rome, Policlinico Umberto I, Viale del Policlinico 155, 00161 Rome, Italycarlo.catalano@uniroma1.it (C.C.); 2 PhD Program in Experimental and Clinical Hepato-Gastroenterology, Department of Radiological, Oncological and Pathological Sciences, Sapienza University of Rome, 00185 Rome, Italy; 3 Vascular and Interventional Radiology Department, Cardarelli Hospital, Via A. Cardarelli 9, 80131 Naples, Italy; francesco.giurazza@aocardarelli.it (F.G.);; 4 Radiology Department, Fondazione IRCCS Cà Granda, Ospedale Maggiore Policlinico, 20122 Milan, Italy; amierardi@yahoo.it (A.M.I.);; 5 Department of Radiology, Santa Chiara Hospital, APSS Trento, Largo Medaglie D’Oro 9, 38122 Trento, Italy; 6 Interventional Radiology Unit, Careggi Hospital, 50134 Florence, Italy; 7 Department of Interventional Radiology, San Camillo Forlanini Hospital, 00152 Rome, Italy; 8 Department of Medical Imaging, University of Cagliari, 09042 Monserrato, Italy; 9 Interventional Radiology Oncology Unit, Deparment of Radiology and Diagnostic Imaging IRCCS Regina Elena National Cancer Institute, 00144 Rome, Italy; 10 Interventional Radiology Unit, Department of Diagnostic Imaging and Oncologic Radiotherapy, Fondazione Policlinico Universitario “Agostino Gemelli”—IRCCS, 00168 Rome, Italy

**Keywords:** DSM-TACE, EmboCept, hepatocellular carcinoma, image-guided therapy, superselective embolization, liver cancer

## Abstract

**Background:** The role of transarterial chemoembolization (TACE) in hepatocellular carcinoma (HCC) management has evolved over recent years. Although it appears that the overall number of procedures is declining, international guidelines now endorse TACE beyond the Barcelona Clinic Liver Cancer (BCLC) intermediate stage, and multiple TACE platforms allow patient-tailored treatments. In this context, degradable starch microspheres TACE (DSM-TACE) may be valuable when the goal is to preserve liver function and spare healthy parenchyma. This study reports multicenter retrospective Italian data to assess the efficacy and safety of DSM-TACE with EmboCept^®^ in patients with early-to advanced-stage HCC, and to evaluate whether procedural selectivity (superselective vs. lobar) influences outcomes. **Methods:** This retrospective multicenter study included 201 patients initially; after applying exclusion criteria, 187 patients (334 HCC nodules) treated across eight centers (2014–2024) were analyzed. Treatment indications were discussed in multidisciplinary tumor boards in all centers. Superselective DSM-TACE was performed in 48 patients (66 nodules, 19.8%), while 139 patients (268 nodules, 80.2%) underwent a lobar approach. Repeated sessions were performed on demand and recorded for lobar treatments. Tumor response was assessed using mRECIST criteria at 1, 3–6, 6–9, and 9–12 months; adverse events were classified according to the Common Terminology Criteria for Adverse Events (CTCAE). efficacy and safety outcomes were compared according to the DSM-TACE approach. **Results:** In terms of safety, analysis confirmed the overall good tolerability of DSM-TACE, with no grade ≥ 3 adverse events and no major complications or procedure-related deaths. No significant differences were observed in post-embolization syndrome (PES) rates between groups. With regard to efficacy, for the entire cohort, the overall response rate (ORR) was 70% at 1 month, 31.6% at 3–6 months, 20.5% at 6–9 months, and 13.5% at 9–12 months, while the disease control rate (DCR) was 91.4% at 1 month, 69% at 3–6 months, 38.6% at 6–9 months, and 27% at 9–12 months. At intermediate follow-up, superselective DSM-TACE achieved higher ORR than lobar treatment at 3–6 months (53.8% vs. 26.4%; *p* = 0.009) and 6–9 months (43.8% vs. 15.3%; *p* = 0.009). Per-nodule analysis confirmed this advantage at 3–6 months (ORR = 66.7% vs. 31.3%; *p* = 0.0008). **Conclusions:** DSM-TACE with EmboCept^®^ provides favorable tumor control and a good safety profile in routine clinical practice. A superselective approach is associated with improved response at intermediate follow-up compared with lobar strategy, supporting DSM-TACE as a flexible therapeutic option for localized HCC.

## Simple Summary

DSM-TACE with EmboCept^®^ was safe and effective in a large Italian multicenter cohort of patients with early- to advanced-stage HCC, showing good tolerability with no major complications. Overall tumor response and disease control were high at early follow-up and gradually declined over time. Importantly, a superselective DSM-TACE approach achieved significantly better tumor response than a lobar strategy at intermediate follow-up, supporting DSM-TACE as a liver-sparing and flexible treatment option, particularly for localized disease.

## 1. Introduction

TACE is considered standard treatment for patients with hepatocellular carcinoma (HCC) in the intermediate stage, according to the Barcelona Clinic Liver Cancer (BCLC) staging system, and is now extended to those in earlier stages who are not suitable candidates for surgery or thermal ablation [1].

DSM-TACE is a technique based on the use of temporary embolic particles that act as a carrier to deliver chemotherapeutic drugs, while creating a temporary block of blood flow, followed by early reperfusion of the treated area [2,3]. The scientific rationale is to preserve the arterial liver supply while optimizing drug accumulation in the tumor and reducing systemic toxicity. For this reason, DSM-TACE is considered a valid and versatile integration to the other well-known TACE options, for example in fragile patients, those with multiple HCC nodules, or those who require lobar/bilobar treatment [4].

Starch microspheres are biocompatible amilomers, derived from hydrolyzed potato starch, and are degraded through enzymatic hydrolysis by alpha-amylase, an enzyme abundantly present in human blood. With a mean diameter of 50 μm, their half-life is usually 40 min depending on body temperature, pH, and amylase serum levels.

Different studies have provided solid evidence of the strong but temporary embolic effect of degradable microspheres, helping to set grounds for their regular use in clinical practice. Both animal and in vivo experiments evaluating embolic properties, time to reperfusion, and histologic changes with degradable starch microspheres showed no histologically detectable differences when comparing pre- and post-embolization parameters [5,6].

Furthermore, in a study comparing VEGF levels during different types of TACE, it was shown that hypoxia after DSM-TACE procedures was not strong enough to produce a VEGF response [7]. This result may have helped the operators build confidence in a platform that favors a short-lived ischemic effect, especially considering that in patients undergoing TACE, high serum VEGF levels are predictive of poor tumor response.

From a technical standpoint, DSM-TACE, like other TACE procedures, is performed using either a transfemoral or transradial approach, but due to the non-standardized nature of the procedure, current administration protocols are subject to variability across centers, and can change according to the administration technique, the degree of selectivity of catheterization, the proposed schedule of repetition, lesion characteristics, etc. In general, the primary goals of our treatments are to deliver the full planned dose of the chemotherapeutic agent and to achieve vascular stasis.

In terms of oncologic efficacy, analysis of the literature on DSM-TACE also shows a high variability in results; this can be attributed to several reasons, such as population heterogeneity, technical features, patient clinical status, and total tumor burden. This variability is reflected in the heterogeneity of oncologic response obtained with DSM-TACE, with overall response rates (ORR) ranging from 84.3% to 39% [8,9,10,11,12,13,14,15,16,17]. In relation to other forms of TACE, a recent study shows no significant differences in terms of procedural efficacy when comparing cTACE, DEB-TACE and DSM-TACE in patients with intermediate-stage HCC treated with palliative intent, while regarding procedural tolerance, patients treated with DSM-TACE showed significantly better outcomes [18]. In fact, one of the main advantages of DSM-TACE is its high safety profile, as well as its role in BCLC-A–D patients ineligible for other treatments [10,17]. In addition, there is recent clinical evidence supporting the efficacy of DSM-TACE in inducing tumor necrosis rates comparable to those of DEB-TACE and cTACE, despite its short embolization time, weakening the belief that prolonged embolization is necessary for achieving substantial tumor necrosis in TACE [19]. Therefore, in the absence of a demonstrated superiority of one technique over another, procedural choice is based on the operators’ preference and according to indications of multidisciplinary tumor boards.

Although DSM-TACE has been widely referenced for years as a promising addition to the current TACE offer with several proven advantages, to date it remains a marginally adopted procedure in clinical practice, with somewhat undefined indications. To help clarify on this point, in 2021 the Cardiovascular and Interventional Society of Europe (CIRSE) released a standard of practice document on all the different types of TACE, to provide technical and clinical guidance; DSM-TACE was fully acknowledged among the TACE options, with well-defined recommendations for its use [20]. In a more recent “think tank” held during the MIOLIVE Conference in 2024 among international experts, it emerged that DSM-TACE was considered mainly for multifocal HCC [21].

With all these considerations made, and given the extensive experience with DSM-TACE of the centers included in our study, the primary aim of our work is to report multicentric retrospective Italian data on the efficacy and safety of DSM-TACE using EmboCept^®^ in patients with early- to advanced-stage HCC; as a secondary objective, we assessed whether procedural differences (superselective vs. lobar approach) influenced outcomes.

## 2. Materials and Methods

### 2.1. Study Design and Setting

The multicenter retrospective cohort involved eight high-volume Italian interventional radiology centers in the period between February 2014 and January 2024. Ethical committee approval was granted (rif.5291), and all patients provided informed consent for the procedure as well as for the anonymized use of their clinical data. Treatment decisions were agreed on in multidisciplinary tumor boards that included hepatologists, oncologists, interventional and diagnostic radiologists, and hepatobiliary/transplant surgeons.

### 2.2. Patient Population

All centers received a dedicated CRF to collect pre-procedural, tumor and patient-specific characteristics. The following parameters were reported in the CRFs provided: baseline laboratory values, baseline tumor number and maximum diameter, and patient characteristics (previous treatments, MELD score, BCLC classification).

After receiving data from 201 patients (160 males, 79.6%), specific exclusion criteria were applied to select eligible patients: absence of follow-up imaging after the DSM-TACE session; liver transplantation immediately after the end of the session; additional locoregional treatment (such as ablation) on liver lesions previously treated with DSM-TACE.

**Figure 1:**
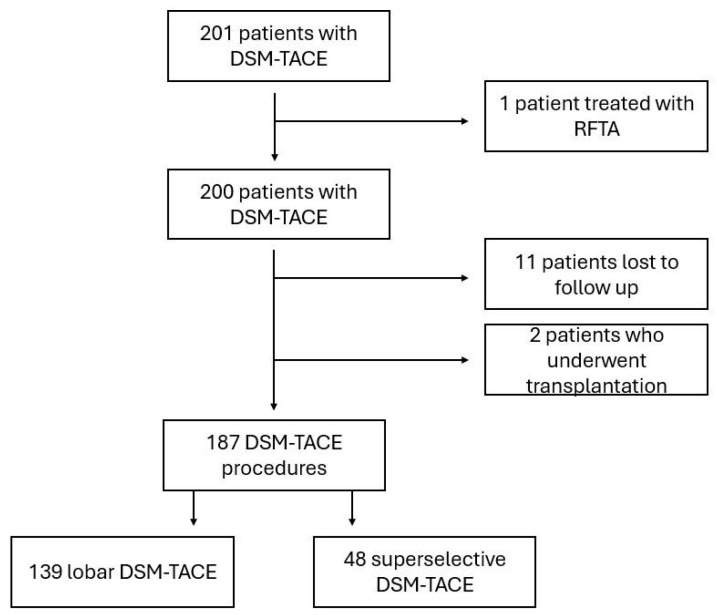
Flow chart showing the selection of procedures with degradable starch microspheres (DSM-TACE) and the final number of DSM-TACE procedures included in the two subgroups.

The final study cohort included 187 patients with 334 HCC nodules (Figure 1). Due to difference in the type of procedure, and to allow for the secondary evaluation to be addressed, they were stratified into two final cohorts based on the degree of treatment selectivity, and categorized as superselective DSM-TACE (48 patients, 66 HCC nodules) and lobar DSM-TACE (139 patients, 268 HCC nodules). Lastly, to verify the oncological outcome, both per-patient and per-nodule analyses were performed.

### 2.3. DSM-TACE Procedure

Procedures were performed under local anesthesia via femoral or radial access, depending on the operator’s preference, using digital subtraction angiography (DSA) and cone beam controlled tomography (CBCT) guidance, as indicated in the CIRSE Standards of Practice (SOP) [20]. DSM-TACE was performed through the “two-step” infusion technique: a drug uptake phase in which 50 mg of Epirubicin in powder diluted in 5 mL of saline was mixed with 4 mL of DSM (EmboCept-S^®^, PharmaCept GmbH, Berlin, Germany) in addition to 15 mL of iodinated contrast agent, and injected intra-arterially, followed by a stop flow phase in which 3.5 mL of pure DSM (EmboCept-S^®^, PharmaCept GmbH, Berlin, Germany) mixed with 6.5 mL of iodinated contrast agent was slowly injected. The procedural endpoint was stasis; in cases in which this was not reached, additional pure Embocept-S was injected. At the end of all procedures, final plane CBCT was acquired to detect proper lesion targeting and coverage.

Procedures were categorized as lobar or superselective (segmental or subsegmental), according to the level of catheterization reached. In cases of lobar administration, repeated sessions were performed on demand. According to the extent and distribution of disease, it was decided to carry out a single lobe administration (two treatments at 4 weeks’ interval), or in cases where tumors were spread bilaterally, a bilobar approach (four treatments, at 2 weeks’ interval) was carried out. It was always recommended to treat the lobe with the larger tumor burden first. This phased approach not only ensured efficient drug absorption in the targeted lesions but was also a means to preserve liver function by avoiding entire liver coverage. All superselective treatment consisted of a single administration.

### 2.4. Oncological Response and Safety Profile

Radiological response for each patient and tumor was measured at 1 month, 3–6 months, 6–9 months and 9–12 months, through contrast-enhanced multidetector computed tomography (MDCT) or contrast-enhanced magnetic resonance imaging (CE-MRI) with hepatobiliary contrast agents.

An experienced radiologist, with over 10 years in CT and MR body imaging, assessed the tumor response based on the modified Response Evaluation Criteria in Solid Tumors (mRECIST).

In order to evaluate the safety profile of DSM-TACE, liver function tests, as well as the occurrence of post embolization syndrome (PES) and other adverse events were recorded following the procedure. PES was characterized by symptoms like fever, nausea, and/or abdominal pain that appeared within 48 h after treatment. Adverse events were classified and graded according to the Common Terminology Criteria for Adverse Events (CTCAE, version 5.0) [22]. Only patients with complete datasets were included for safety evaluations.

### 2.5. Statistical Analysis

The Kolmogorov–Smirnov Z test was used for assessing normality distribution. Continuous variables were reported as mean ± standard deviation or as median with a 95% confidence interval (CI), depending on what was appropriate. For paired laboratory data (pre- vs. post-treatment), we used either Student’s *t*-test or the Wilcoxon signed-rank test based on data distribution.

We compared oncological responses (mRECIST) between groups using the Chi-square test at four follow-up time points: 1 month, 3–6 months, 6–9 months and 9–12 months, applying Bonferroni correction for any post hoc analyses to both the patients’ and nodules’ data. The data were stratified according to BCLC using the Chi-square test in 3 × 4 tables for each of the time points.

Laboratory analysis adverse events were categorized accordingly to the grade defined by CTCAE version 5.0 [22]. The analysis for the comparison between lobar and superselective embolization was categorized into two groups, ≥1 and ≥2, using the Chi-square test.

To identify predictors of objective response at 9–12 months, we relied on a logistic regression model that included factors like MELD-Na, gender, age, alpha-fetoprotein (AFP), and maximum tumor diameter. We also conducted intergroup comparisons of fold changes in laboratory values and safety outcomes (lobar vs. superselective DSM-TACE) using either Student’s *t*-test or Mann–Whitney U test, as appropriate. All analyses were carried out using MedCalc 18.2.1 (MedCalc Software, Ostend, Belgium). We defined statistical significance as a *p*-value of less than 0.05 (two-tailed).

## 3. Results

### 3.1. Study Population

**Figure 2:**
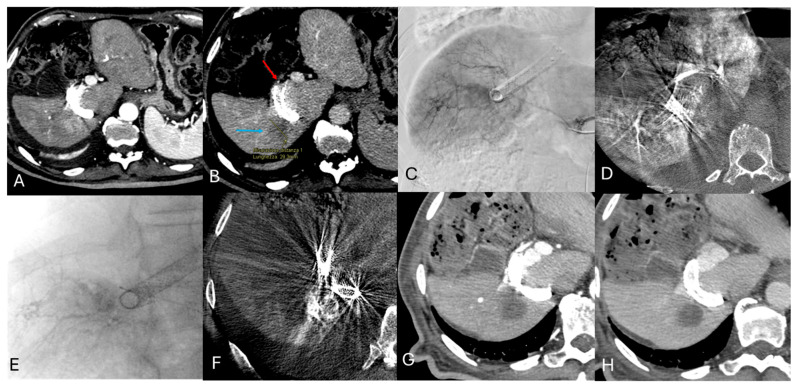
76 yo, male, Child B8 cirrhosis with a 3 cm HCC nodule, unfit for ablation for its adjacency to a Transjugular Intrahepatic Portosystemic Shunt (TIPS) procedure. (**A**) Arterial phase shows 3 cm hypervascular HCC nodule (VIIs-VIs) with *wash-in* features and (**B**) early *wash-out* in nephrogenic phase (blue arrow); the nodule is posterior to the TIPS (red arrow). (**C**) Pre-operative work-up: non-selective DSA is used to assess the hepatic lobar arterial anatomy and identify which lobar/segmental branches supply tumor feeders. (**D**) Pre-operative CBCT arterial phase. (**E**) Selective angiography via coaxial microcatheter confirms tumor enhancement. In the 2 phases of DSM-TACE, 4 mL Embocept + Epirubicin + contrast, and the remaining 3.5 mL Emobocept + contrast, were injected until stop flow. (**F**) Immediate post-procedural non-enhanced CBCT demonstrates that nodule is totally covered and opacified with embolics. (**G**,**H**) Arterial and venous phase of 1-month CT scan shows lesion’s complete response (CR) with a single superselctive DSM-TACE session.

A total of 201 patients diagnosed with HCC were initially included in the study. After excluding those who did not meet the criteria, the final group included 187 patients with 334 HCC nodules. Of these, 48/187 patients (with 66 HCCs, accounting for 19.8%; median of 1.4 lesions per patient) underwent superselective DSM-TACE (Figure 2), while 139/187 patients (with 261 HCCs, making up 80.2%; median of 1.9 lesions per patient) received a lobar approach. The baseline demographic and clinical characteristics were similar across both groups, a summary of which can be found in Table 1 and Table 2.

### 3.2. Safety Profile

The safety profile analysis confirmed the overall good tolerability of DSM-TACE treatment, with no grade ≥ 3 adverse events and no major complications or procedure-related deaths. No statistically significant differences were observed between the two approaches for any of the main blood chemistry parameters (AST, ALT, bilirubin, GGT, creatinine, platelets) (all *p* > 0.05). All clinical events were grade ≤ 2 and resolved spontaneously or with standard symptomatic therapy.

Grade 1 events were observed in 39% (40/102) of cases for AST, 59% (60/102) for ALT and 22% (22/102) for total bilirubin in the lobar group, compared with 67% (6/9), 73% (7/9) and 13% (1/9) in the superselective group (*p* = 0.11, 0.21 and 0.39, respectively). Grade 2 alterations occurred in 19% (19/102) of lobar patients for AST and in 4% (4/102) for ALT, compared to 6% (1/9) and 11% (1/9) in the superselective group (*p* = 0.16 and 0.32, respectively). Overall, grade 1–2 laboratory abnormalities resolved spontaneously by the next follow-up, with no clinical signs of liver damage and no need for additional medical treatment (Table 3).

PES occurred in 42/144 patients (29.2%), with a comparable distribution between the lobar group (36/110; 32.7%) and the superselective group (6/34; 17.6%), *p* = 0.14. The main symptoms associated with PES were abdominal pain, nausea, and vomiting. Abdominal pain was reported in 37/145 patients (25.5%), divided into 31/110 (28.2%) in the lobar group and 6/35 (17.1%) in the superselective group (no significant difference, with *p* = 0.28). Nausea and vomiting occurred in 2/144 (1.38%) of the total cases, with no significant differences between the two approaches (0/110 (0%) vs. 2/34 (5.9%), *p* = 0.53 for both), as shown in Table 4.

### 3.3. Efficacy

The entire cohort oncological response at 1 month showed an overall response rate (ORR) of 70% (130/187) and a disease control rate (DCR) of 91.4% (171/187). At 3–6 months after treatment, the ORR was 31.6% (43/136) and the DCR was 69% (94/136). At 6–9 month follow-up, ORR was 20.5% and DCR was 38.6%; at 9–12 months, ORR and DCR were respectively 13.5% and 27%.

### 3.4. Per-Patients Oncological Response

The per-patient analysis highlighted differences between the two techniques of administration (lobar vs. superselective), particularly in the intermediate assessments.

There was a significant trend towards an improved response at 3–6 months if the DSM-TACE was administered in a selective vs. non-selective manner: at 3–6 months, complete response (CR) was 26.9% (7/26) vs. 7.3% (8/110); while partial response (PR) was 26.9% (7/26) vs. 19.1% (21/110), with *p* = 0.026.

The advantage of superselective vs. lobar approach was also maintained in the 6–9 months following the procedure, with a CR of 18.75% (3/16) vs. 2.8% (2/72) and a PR of 25% (4/16) vs. 12.5% (9/72), with *p* = 0.05 (Table 5). However, this result loses its statistical significance when stratified for BCLC (Table 6).

### 3.5. Per-Nodule Oncological Response

In the per-nodule analysis, conducted on 334 lesions (268 treated with a lobar approach and 66 with a superselective approach), the efficacy of DSM-TACE showed significant differences between the two technical strategies, especially in the intermediate follow-up.

The largest difference emerged at the 3–6-month follow-up, when the superselective approach achieved a significantly higher oncological response: ORR was 66.7% (16/24) compared to 31.3% (31/99) for the lobar approach (*p* = 0.0008). DCR followed the same trend, with values of 91.7% (22/24) in the superselective group vs. 70.7% (70/99) in the lobar group. (Table 7).

## 4. Discussion

This multicenter retrospective study first evaluated the oncological efficacy and safety of DSM-TACE in patients with HCC and secondarily focused on the impact of catheter selectivity on treatment outcomes. By comparing lobar and superselective approaches in a real-world clinical setting, our analysis demonstrates that the degree of selectivity represents a relevant technical determinant of oncological response, particularly at intermediate follow-up, while maintaining a comparable safety profile.

In terms of efficacy relative to the entire cohort, oncological response at 1 month showed an ORR of 70% and a DCR of 91.4%. At 3–6-month follow-up, the ORR was 31.6% and DCR was 69%. At 9–12 months, ORR and DCR dropped respectively to 13.5% and 27%, a trend consistent with the palliative nature of TACE and the natural history of HCC. When performing a sub analysis, our results show that patients treated with a superselective approach achieved statistically significantly higher ORR and DCR at 3–6 months, compared to those undergoing lobar DSM-TACE (ORR 53.8% vs. 26.4%; DCR 73% vs. 68.2%). This advantage persisted at 6–9 months, although differences tended to attenuate over time, as typically occurs for TACE treatments, where there is a high initial response followed by a progressive decline in the medium term. These findings suggest that technical refinement, rather than the embolic agent itself, plays a central role in modulating treatment efficacy. The observed response rates fall within the upper range of those reported in the literature for DSM-TACE, where considerable heterogeneity has been described, with ORRs ranging from approximately 40% to over 85%, and DCRs between 60% and 90% [9,10,11,13].

In this context, if we compare our study to the work of Collettini et al. in HepaStar [17], interestingly, the early response observed in our cohort is higher, with a 1-month ORR of 70% compared to 27% in HepaStar, suggesting greater initial treatment efficacy. Although a stratified analysis based on the degree of selectivity was not reported in their study, the prevalence of lobar procedures (approximately two-thirds of cases) reflects a similar technical case series to our lobar group, reinforcing the reliability of the comparison. The procedural stratification in our study highlights an improvement in response in the patients treated superselectively; overall, these elements make our work complementary to HepaStar and offer new evidence on the importance of technical accuracy in determining the efficacy of DSM-TACE.

In general, the variability we find in the literature is likely a reflection of the differences in real-life clinical settings. Factors such as patient selection, tumor burden/disease stage, level of catheterization (lobar vs. segmental or subsegmental), volume and concentration of microspheres and drugs, etc., all significantly influence treatment response and tolerability [17,23,24,25,26]. Therefore, the wide variability of outcomes reported for DSM-TACE is probably due to procedural heterogeneity rather than intrinsic limitations of the microspheres themselves. As per other TACE techniques, our data support the hypothesis that distal, superselective delivery of the chemoembolic mixture enhances intratumoral drug concentration and embolic homogeneity, while limiting non-target embolization and preserving surrounding healthy liver parenchyma. The versatility of DSM-TACE, including its compatibility with different chemotherapeutic drugs and its temporary embolic effect, may further contribute to this variability when standardized protocols are not applied.

As previously mentioned, current CIRSE Standards of Practice [20] offer guidance on the use of DSM-TACE also based on disease extent and liver function. These recommendations, based on European expert consensus, emphasize the importance of procedural standardization and appropriate sequencing in bilobar disease. Within this framework, our findings suggest that in selected patients, particularly those with limited or localized tumor burden, a superselective strategy may provide superior short- to medium-term tumor control, without increasing the risk of complications, and propose a broader indication for DSM-TACE.

This concept is further reinforced by the per-nodule analysis performed in our study, which confirms a marked oncological advantage of the superselective approach at intermediate follow-up. At 3–6 months, superselective DSM-TACE achieved an ORR of 66.7% vs. 31.3% for lobar treatment, along with a higher DCR (91.7% vs. 70.7%). These results align with previous evidence indicating that selective catheterization is a key determinant of both efficacy and hepatic toxicity in TACE, regardless of whether conventional, drug-eluting beads, or temporary embolic platforms are used [24,27,28,29,30]. By enabling a more uniform penetration of the chemotherapeutic agent into the tumor microvasculature, superselective delivery may optimize tumor necrosis while minimizing collateral damage.

With regard to safety, the results obtained on the entire cohort confirmed the overall good tolerability of DSM-TACE, with no grade ≥ 3 adverse events and no major complications or procedure-related deaths. When performing the sub analysis between the two groups, both technical approaches demonstrated a favorable safety profile. The occurrence of PES (approximately 30%), as well as nausea, vomiting, and abdominal pain (<3%), was consistent with rates reported in large published series, which place the incidence of PES between 4% and 35% [10,15]; no significant differences were observed between lobar and superselective treatments. Laboratory abnormalities were transient and mild, with no increase in clinically relevant liver toxicity. These findings confirm that DSM-TACE is well tolerated even when performed in a lobar fashion, likely due to the temporary vascular occlusion and the rapid enzymatic degradation of starch microspheres which allow early reperfusion of treated liver segments.

Consistently to what can and should be expected when performing intrarterial treatments in general, regardless of the device used, studies comparing DSM-TACE to other TACE platforms [16,18,25] have shown similar ORR and DCR, in line with those obtained in the present study, supporting the view that DSM-TACE represents a valid alternative. Although repeatedly stated in the literature, the choice of one TACE over another remains an operator-dependent preference, the consistency of our results with existing data strongly suggest that treatment selectivity is a key technical factor.

Taken together, our results support the concept that technical optimization—rather than prolonged embolization, more aggressive ischemia, or different choice of embolic—is sufficient to achieve meaningful tumor control, while preserving a healthy liver. Nevertheless, this study has limitations inherent to its retrospective design, including potential selection bias, heterogeneity in procedural techniques across centers, and the absence of a centralized imaging review process. Additionally, although the imbalance between lobar and superselective groups reflects real-world practice, it may have limited statistical power in some subgroup analyses. Prospective, multicenter studies with standardized technical protocols and predefined treatment algorithms are warranted to confirm these observations and to better define the optimal role of DSM-TACE within the multidisciplinary management of HCC.

## 5. Conclusions

Our results confirm that DSM-TACE is a safe and effective procedure, regardless of the degree of catheter selectivity; however, it highlights an oncological advantage of the superselective approach in the medium-term follow-up. In fact, patients treated in a superselective manner showed a consistent trend towards higher response and disease control rates at intermediate follow-up. This strategy appears particularly beneficial in patients with limited disease, where it can offer an optimal tradeoff between therapeutic efficacy and overall safety. In line with the principles of precision for medicine and personalized treatments, our data underscores the crucial role of technical and procedural standardization in maximizing therapeutic efficacy while preserving liver function.

## Figures and Tables

**Table 1:** Demographic characteristics of the entire cohort; SD, standard deviation; M, male; F, female; BCLC Barcelona Clinic Liver Cancer; HCV Hepatitis C virus; HBV Hepatitis B virus; NASH Non-alcoholic steatohepatitis; MELD Model for End-Stage Live Disease; several patients presented multiple aetiologies; percentages are calculated on the total number of patients (***n*** = 187).

Variable	Gruppo 0 (*N* = 139)	Gruppo 1 (*N* = 48)	*p*-Value
Age	69.76 ± 9.59	68.89 ± 9.64	8.53
Score Child	6.07 ± 1.20	5.24 ± 1.15	<0.001
MELD	8.88 ± 1.97	9.74 ± 2.78	2.21
MELDNa	9.88 ± 2.71	10.54 ± 3.07	7.22
Sex			4.24
F	24 (17.6%)	12 (25.0%)	
M	115 (82.4%)	36 (75.0%)	
Performance status			10.00
0	75 (54.3%)	28 (57.8%)	
1	54 (39.1%)	19 (39.1%)	
2	10 (6.5%)	1 (3.1%)	
Child-Pugh			0.10
A	94 (67.6%)	42 (87.3%)	
B	41 (29.6%)	6 (12.7%)	
C	4 (2.8%)	0 (0.0%)	
BCLC			0.16
A	76 (54.9%)	31 (64.1%)	
B	53 (38.0%)	10 (20.3%)	
C	10 (7.0%)	7 (15.6%)	
Etiology			0.04
Alcohol	55 (39.5%)	12 (25.6%)	
HCV	47 (33.6%)	19 (39.5%)	
NASH	16 (11.8%)	15 (30.2%)	
HBV	16 (11.8%)	0 (0.0%)	
Other	5 (3.3%)	2 (4.7%)	

**Table 2:** Baseline characteristics of the entire cohort.

Category	Subcategory	*N* = 187
Portal vein tumor thrombosis	Yes	19 (9.5%)
	No	182 (90.5%)
Previous curative treatments	None	
Tumor burden	Unilobar	149 (74.12%)
	Bilobar	52 (25.8%)
Tumor load	Solitary	47 (23.4%)
	Bifocal	24 (12%)
	Multifocal	130
Largest tumor size (mm)	Mean ± SD	32.9 ± 21.4
	Median (IQR)	28 (35–21)

**Table 3:** Treatment-related adverse events by CTCAE grade; CTCAE = Common Terminology Criteria for Adverse Events (version 5.0).

Parameter Grade 1	Lobar DSM-TACE (n/N, %)	Superselective DSM-TACE (n/N, %)	*p* Value
AST ↑	40/102 (39%)	6/9 (67%)	0.11
ALT ↑	60/102 (5–9%)	7/9 (73%)	0.21
Total bilirubin ↑	22/102 (22%)	1/9 (13%)	0.39
GGT ↑	6/102 (6%)	0/9 (0%)	0.45
Creatinine ↑	2/102 (2%)	0/9 (0%)	0.64
Platelets ↓ **Grade 2**	7/102 (7%)	1/9 (5%)	0.76
AST ↑	19/102 (19%)	1/9 (6%)	0.16
ALT ↑	4/102 (4%)	1/9 (11%)	0.32
Total bilirubin ↑	3/102 (3%)	0/9 (0%)	0.49
GGT ↑	1/102 (1%)	0/9 (0%)	0.68
Creatinine ↑	1/102 (1%)	0/9 (0%)	0.74

**Table 4:** Treatment-related AEs. PES: Post-embolization syndrome.

PES	Yes	Grades	χ^2^	*p* =
Lobar DSM-TACE	36/110 (32.7%)	1–2	2.176	0.1402
Superselective DSM-TACE	6/34 (17.6%)	1–2		
**Nausea**				
Lobar DSM-TACE	0/110 (0%)	0	0.389	0.5329
Superselective DSM-TACE	1/34 (2.9%)	1		
**Pain**				
Lobar DSM-TACE	31/110 (28.2%)	1–2	1.171	0.2792
Superselective DSM-TACE	6/35 (17.1)	1–2		
**Vomit**				
Lobar DSM-TACE	0/110 (0%)	0	0.389	0.5329
Superselective DSM-TACE	1/34 (2.9%)	1		

**Table 5:** Per-patient oncological Response. Chemoembolization with Degradable Starch Microspheres. Bonferroni’s correction in this case significantly addressed *p* at 0.026.

	Entire Cohort	Lobar DSM-TACE	Superselective DSM-TACE	χ^2^	*p* =
**1-month**					
CR	28/187 (15%)	18/139 (12.9%)	10/48 (20.8%)		
PR	102/187 (54.5%)	74/139 (53.2%)	28/48 (20.14%)	6.62	0.085
SD	41/187 (22%)	35/139 (25.2%)	6/48 (4.3%)		
PD	16/187 (8.6%)	12/139 (8.6%)	4/48 (8.3%)		
ORR (CR + PR)	130/187 (70%)	92/139 (66.2%)	38/48 (79.2%)	2.34	0.126
DCR (CR + PR + SD)	171/187 (91.4%)	127/139 (91.4%)	44/48 (91.7%)	0.002	0.96
**3–6 months**					
CR	15/136 (11%)	8/110 (7.3%)	7/26 (26.9%)		
PR	28/136 (20.6%)	21/110 (19.1%)	7/26 (26.9%)	9.27	0.026
SD	51/136 (37.5%)	46/110 (41.8%)	5/26 (19.2%)		
PD	46/136 (33.8%)	39/110 (35.5%)	7/26 (26.9%)		
ORR (CR + PR)	43/136 (31.6%)	29/110 (26.4%)	14/26 (53.8%)	6.77	0.009
DCR (CR + PR + SD)	94/136 (69%)	75/110 (68.2%)	19/26 (73%)	0.23	0.63
**6–9 months**					
CR	5/88 (5.7%)	2/72 (2.8%)	3/16 (18.75%)		
PR	13/88 (14.8%)	9/72 (12.5%)	4/16 (25%)	7.82	0.050
SD	16/88 (18.2%)	13/72 (18.1%)	3/16 (18.75%)		
PD	54/88 (61.4%)	48/72 (66.7%)	6/16 (37.5%)		
ORR (CR + PR)	18/88 (20.5%)	11/72 (15.3%)	7/16 (43.75%)	6.72	0.009
DCR (CR + PR + SD)	34/88 (38.6%)	24/72 (33.3%)	10/16 (62.5%)	5.62	0.018
**9–12 months**					
CR	2/37 (5.4%)	1/29 (3.4%)	1/8 (12.5%)		
PR	3/37 (8%)	3/29 (10.3%)	0 (0%)	0.87	0.83
SD	5/37 (13.5%)	4/29 (13.8%)	1/8 (12.5%)		
PD	25/37 (67.6%)	20/29 (69%)	5/8 (62.5%)		
ORR (CR + PR)	5/37 (13.5%)	4/29 (13.8%)	1/8 (12.5%)	0.009	0.92
DCR (CR + PR + SD)	10/37 (2.7%)	8/29 (27.6%)	2/8 (25%)	0.01	0.92

**Table 6:** Oncological response stratified according to BCLC.

BCLC	CR	PR	SD	PD
**A**	0 (0.0%)	0 (0.0%)	4 (20%)	16 (80%)
**B**	3 (20%)	2 (13.3%)	1 (6.7%)	9 (60%)
**C**	0 (0.0%)	1 (33.3%)	0 (0.0%)	2 (66.7%)
χ^2^	11.28			
*p* =	0.08			

**Table 7:** Per-nodule oncological response. Bonferroni’s correction in this case significantly addressed *p* at 0.0008.

	Lobar DSM-TACE	Superselective DSM-TACE	χ^2^	*p* =
**1 month**			0.565	0.9043
CR	61/268 (22.8%)	13/66 (19.7%)		
PR	129/268 (48%)	34/66 (51.5%)		
SD	62/268 (23%)	16/66 (24.2%)		
PD	16/268 (6%)	3/66 (4.5%)		
ORR	190/268 (70.9%)	47/66 (71.2%)		
DCR	252/268 (94%)	63/66 (95.5%)		
**3–6 months**			16.836	0.0008
CR	8/99 (8.1%)	9/24 (37.5%)		
PR	23/99 (23.2%)	7/24 (29.2%)		
SD	39 (39.4%)	2/24 (8.3%)		
PD	23 (23.2%)	6/24 (25%)		
ORR	31/99 (31.3%)	16/24 (66.7%)		
DCR	70/99 (70.7%)	22/24 (91.7%)		
**6–9 months**			6.458	0.0913
CR	5/56 (8.9%)	4/16 (25%)		
PR	9/56 (16.1%)	4/16 (25%)		
SD	9/56 (16.1%)	4/16 (25%)		
PD	33/56 (58.9%)	4/16 (25%)		
ORR	14/56 (25%)	8/16 (50%)		
DCR	23/56(41%)	12/16 (75%)		
**9–12 months**			0.507	0.9174
CR	3/26 (11.5%)	1/5 (20%)		
PR	1/26 (3.8%)	0/0 (0%)		
SD	7/26 (26.9%)	1/5 (20%)		
PD	15/26 (57.7%)	3/5 (60%)		
ORR	4/26 (15.4%)	1/5 (20%)		
DCR	11/26 (42.3%)	2/5 (40%)		

## Data Availability

The data presented in this study are available on request from the corresponding author due to privacy issues.
